# Case Report: Response to crizotinib treatment in a patient with advanced non-small cell lung cancer with *LDLR-ROS1* fusion

**DOI:** 10.3389/fonc.2023.1169876

**Published:** 2023-04-20

**Authors:** Yun Shu, Zhouyu Wang, Hongjuan Shang, Wei Le, Yan Lei, Longzhang Huang, Liming Tao, Jun Chen, Jing Li

**Affiliations:** ^1^ Department of Medical Oncology, Third People’s Hospital of Jiujiang City, Jiujiang, China; ^2^ Department of Medical Affairs, Berry Oncology Corporation, Beijing, China; ^3^ Fujian Key Laboratory of Advanced Technology for Cancer Screening and Early Diagnosis, Fuzhou, China

**Keywords:** advanced non-small cell lung cancer, c-ros oncogene 1, *LDLR-ROS1* fusion, crizotinib, next-generation sequencing

## Abstract

C-ros oncogene 1 (*ROS1*) fusion is a pathogenic driver gene in non-small cell lung cancer (NSCLC). Currently, clinical guidelines from the National Comprehensive Cancer Network (NCCN) have recommended molecular pathologic tests for patients with NSCLC, including the detection of the *ROS1* gene. Crizotinib is a small molecule tyrosine kinase inhibitor of anaplastic lymphoma kinase (*ALK*), *ROS1*, and mesenchymal-epithelial transition (*MET*). In recent years, the efficacy of crizotinib in NSCLC patients with *ROS1* fusion has been reported. Here, a 77-year-old woman was diagnosed with stage IVA lung adenocarcinoma harboring a novel low-density lipoprotein receptor (*LDLR*)*-ROS1* fusion variant. This novel *LDLR-ROS1* fusion was identified by targeted DNA next-generation sequencing (NGS) panel and then verified by RNA fusion panel based on amplicon sequencing. This patient benefited from subsequent crizotinib therapy and achieved progression-free survival of 15 months without significant toxic symptoms. Our case report recommended a promising targeted therapeutic option for patients with metastatic NSCLC with *LDLR-ROS1* fusion and highlighted the importance of genetic testing for accurate treatment.

## Introduction

Lung cancer is one of the most common malignancies, both in terms of morbidity and mortality worldwide, among which non-small cell lung cancer (NSCLC) accounts for most cases (1, 2). Most patients with NSCLC have poor prognosis. Advances in molecular pathology and targeted therapy have changed the prognosis of NSCLC (3). Until now, driver genes such as *EGFR*, *ALK*, *ROS1*, *MET*, and *RET* have led to a variety of changes in NSCLC.

C-ros oncogene 1 (*ROS1*) gene is located at 6q22, on the long arm of chromosome 6, encoding a receptor tyrosine kinase in the subclass of the insulin receptor family (4, 5). Since 2007, researchers have revealed that multiple partner genes fuse with the 3′ *ROS1* fragment containing the intact tyrosine kinase domain. The expression of these genes results in autophosphorylation of the *ROS1* tyrosine kinase, which activates and triggers survival signaling pathways, driving malignant cell proliferation (6). Ultimately, *ROS1* fusion has been identified as a pathogenic driver gene in NSCLC, with an incidence of 1%–2% (7). Patients with *ROS1* fusion have distinct clinical features such as younger age, no or slight smoking history, and histology of adenocarcinoma (7, 8). Many unknown fusions and other oncogenic mutations can be detected by next-generation sequencing (NGS), and new *ROS1* fusion partner genes have been identified in lung cancer (9). Interestingly, the tyrosine kinase domain of *ROS1* is structurally similar to anaplastic lymphoma kinase (*ALK*), another molecular driver of NSCLC (10). Considering the structural similarity, some *ALK* tyrosine kinase inhibitors, such as crizotinib, are also active against *ROS1 (* 11). Crizotinib and entrectinib have been approved to treat *ROS1* fusion-positive NSCLC (12). Crizotinib has received approval in 70 countries worldwide for the treatment of *ROS1*-positive patients with advanced NSCLC. CD74 is the most common *ROS1* fusion partner (13). In addition, several novel fusion partners have been discovered in NSCLC, such as FIG, SLC34A2, and SDC4 (14). However, the duration of response may vary among patients with different clinical and genetic characteristics.

In this case, we reported a novel *LDLR-ROS1* fusion variant that was identified in a patient diagnosed with advanced lung adenocarcinoma. The patient received crizotinib treatment and exhibited an evident response.

## Case presentation

**Figure 1:**
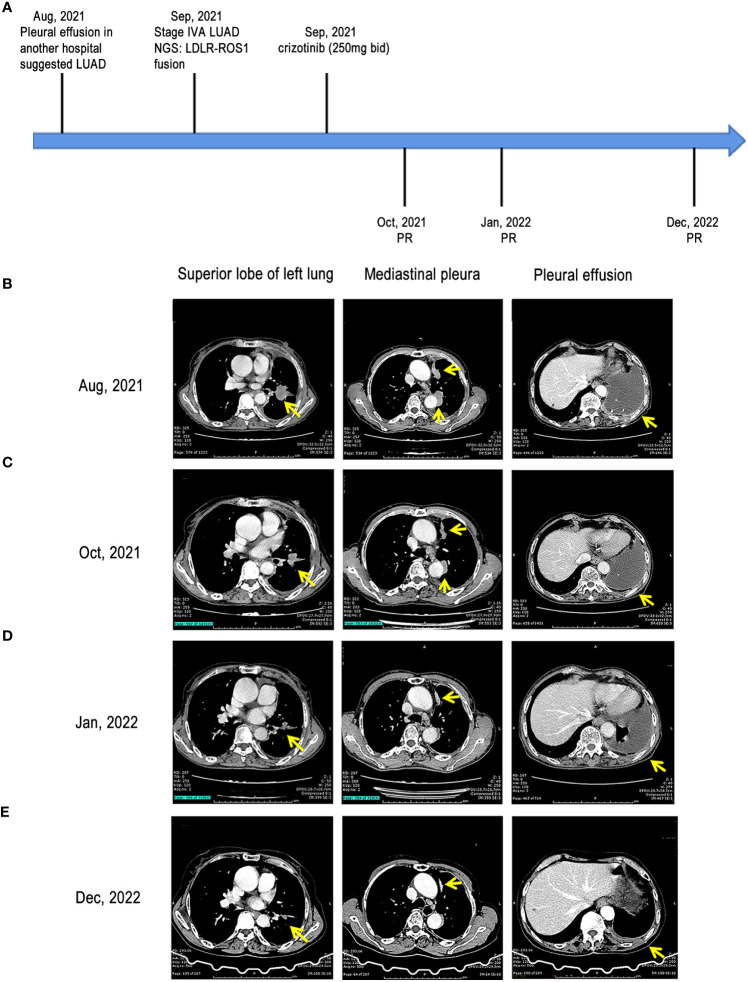
Treatment milestones of our case. **(A)** treatment timeline of the case; **(B)** CT scans of the left upper lobe mass, mediastinal pleural nodule, and pleural effusion at baseline; **(C)** CT scans of the left upper lobe mass, mediastinal pleural nodule, and pleural effusion at the first follow-up after treatment; **(D)** CT scans of the left upper lobe mass, mediastinal pleural nodule, and pleural effusion at the second follow-up after treatment; **(E)** CT scans of the left upper lobe mass, mediastinal pleural nodule, and pleural effusion at the third follow-up after treatment LUAD, lung adenocarcinoma; CT, computed tomography; *LDLR*, low-density lipoprotein receptor; *ROS1*, *ROS* proto-oncogene 1; PR, partial response.

A 77-year-old woman with no smoking history presented with cough, chest tightness, and other symptoms since May 2021. The patient visited the Lushan People’s Hospital on August 15, 2021. A plain chest computed tomography (CT) scan revealed a mass, pleural nodule, and pleural effusion in the left lung. Lung adenocarcinoma was confirmed by pleural effusion cytology after closed thoracic drainage (**Figure 1A**). Subsequently, targeted-NGS sequencing based on pleural effusion was performed, but no gene mutations associated with targeted therapy were detected. After cis-platinum pleural perfusion, she was transferred to the Third People’s Hospital of Jiujiang on August 30, 2021.

**Figure 2:**
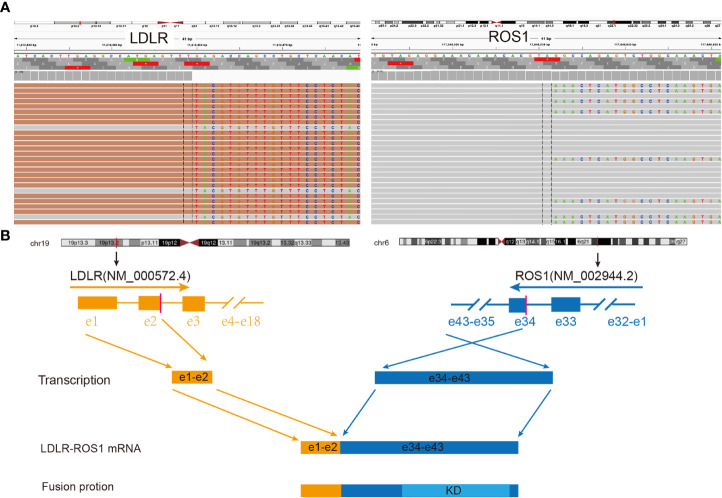
Identification and verification of *LDLR-ROS1* fusion in this case. **(A)** RNA sequencing reads indicating visualization of the *LDLR* and *ROS1* fusion regions using the Integrative Genomics Viewer (IGV) software. The fusion breakpoints are localized at chr19: p13.2: 11,212,559 and chr6: q22.1: 117,646,031, respectively; **(B)** Schematic of genomic rearrangement involving the fusion breakpoints at mRNA and protein levels; the transcript resulted in exons 1–2 of *LDLR* fused to exons 34–43 of *ROS1*, including kinase domain. *LDLR*, low-density lipoprotein receptor; *ROS1*, *ROS* proto-oncogene 1; Orange color represents *LDLR*; Blue color represents *ROS1*; KD, kinase domain; e, exon.

After admission, the patient underwent further examinations as follows: 1) the specific tumor marker carcinoembryonic antigen (CEA) level was normal (3.52 µg/L); 2) an enhanced CT scan further revealed a mass in the left upper lobe of the lung and left pleural thickening with nodular formation (**Figure 1B**); 3) magnetic resonance imaging (MRI) of the head revealed no obvious abnormality; 4) single photon emission CT/CT (SPECT/CT) scan exhibited no bone metastasis. Subsequently, left pleural metastatic adenocarcinoma was diagnosed by biopsy pathology (CT2N2M1a, IVA). A few days later, the formalin-fixed paraffin-embedded (FFPE) tissue was examined using the targeted DNA NGS panel (Berry Oncology Corporation, Beijing, China) and a rare low-density lipoprotein receptor (*LDLR*)-*ROS1* fusion variant was identified. Besides, an RNA fusion panel based on amplicon sequencing (Berry Oncology Corporation, Beijing, China) was also used to verify this fusion variant from total RNA which isolated from FFPE tissues. Both the DNA and RNA-based sequencing technology revealed *LDLR* exon 2-*ROS1* exon 34 rearrangement in the tissue (**Figure 2**). This *LDLR-ROS1* fusion retained the kinase domain of *ROS1*, which could cause constitutive kinase activity and oncogenic transformation.

Based on these genetic testing results, the patient was orally administered 250 mg of crizotinib twice a day (250 mg bid) since September 10, 2021. After a few days, the patient noticed an improvement in symptoms. Subsequently, she was discharged from the hospital and continued taking medication. One month later (October 2021), the patient visited the hospital for further review. The objective response was evaluated according to the Response Evaluation Criteria in Solid Tumors (RECIST 1.1) (15). Chest CT scan revealed that left lobe mass and mediastinal pleural nodules reduced, and pleural effusion was absorbed, indicating that this patient achieved partial response (PR) (**Figure 1C**). In addition, the patient presented no discomforting symptoms such as cough and chest distress. In January 2022, the chest CT scan demonstrated that the lesion shrank further, pleural effusion continued to decrease, and PR was noted (**Figure 1D**). Another chest CT scan conducted in December 2022 demonstrated that the lesion shrank further, pleural effusion elementally disappeared, and the treatment showed PR (**Figure 1E**).

## Discussion

In this case, we reported an infrequent *LDLR-ROS1* fusion in the patient, who was an elderly woman of Asian origin without a smoking history, diagnosed with lung adenocarcinoma. The pathogenic driver gene was *ROS1*, and the fusion included exons 34–43 of *ROS1* that retained the complete kinase domain. Once activated, ROS1 signaling promotes malignant cell growth through a series of downstream pathways, ultimately leading to cancer formation (16).

Initially, crizotinib, a *MET/ALK* multi-targeted receptor tyrosine kinase inhibitor, was approved for the treatment of *ALK*-rearranged NSCLC (17). The ATP-binding sites of kinase domains share 77% amino acid identity between the *ALK* and *ROS1* genes (10). Furthermore, crizotinib has a high affinity for *ROS1*, which further effectively inhibits *ROS1* signaling and cell viability in cell lines expressing *ROS1* fusions (18).

The fusion partner (*LDLR* gene) located on chromosome 19 (19p13), consisting of 18 exons, plays a pivotal role in cholesterol homeostasis and lipid metabolism under normal physiological conditions (19, 20). Targeted-NGS sequencing based on pleural effusion at Lushan People’s Hospital did not detect an ROS-1 fusion, while targeted-DNA panel and RNA fusion panel based on needle biopsy samples at our hospital detected an ROS-1 fusion. The sensitivity of NGS based on pleural effusion for detecting actionable mutations was found to be lower than that based on tumor tissues (21, 22). In this case, the fusion included exons 1–2 of the *LDLR* gene and exons 34–43 of the *ROS1* gene. This fusion includes kinase domains that should theoretically be sensitive to targeted therapy. It remains unclear whether patients with advanced NSCLC and *LDLR-ROS1* fusion can benefit from the treatment of crizotinib. In our case, crizotinib was selected as the treatment for the patient diagnosed with advanced NSCLC with *ROS1* fusion. One month later, evident changes were observed on imaging, confirming surprising therapeutic effects, and the clinical response to crizotinib continued for at least 15 months during follow-up.

In another case report, adjuvant crizotinib provided a favorable survival benefit in a patient with resectable stage IIIA NSCLC with the *LDLR-ROS1* fusion (23). In this case, postoperative targeted therapy exhibited promising efficacy, and the patient’s clinical and radiological follow-ups revealed no evidence of progression or recurrence, with a relapse-free survival of more than 29 months. Our case demonstrated the use of crizotinib as the treatment in a patient with metastatic NSCLC with fusion, and the patient experienced progression-free survival of 15 months.

## Conclusion

Our study provided clinical evidence that advanced NSCLC patients harboring *LDLR-ROS1* fusion may have durable responses to crizotinib. Crizotinib may be an effective treatment option for patients with advanced NSCLC with *LDLR-ROS1* fusion.

## Data availability statement

The original contributions presented in the study are included in the article/supplementary material. Further inquiries can be directed to the corresponding authors.

## Ethics statement

The studies involving human participants were reviewed and approved by Ethical Committee of Medical Research, Third People’s Hospital of Jiujiang City. The patients/participants provided their written informed consent to participate in this study. Written informed consent was obtained from the individual(s) for the publication of any potentially identifiable images or data included in this article.

## Author contributions

HS and WL contributed to patient management. YS and JL designed and reviewed the report. YS wrote the manuscript. ZW, LT, and JC reviewed and corrected the manuscript. LH, ZW and YL drew the pictures. All authors contributed to the article and approved the submitted version.
